# Coronary and carotid artery dysfunction and K_V_7 overexpression in a mouse model of Hutchinson-Gilford progeria syndrome

**DOI:** 10.1007/s11357-023-00808-3

**Published:** 2023-05-26

**Authors:** Álvaro Macías, Rosa M. Nevado, Cristina González-Gómez, Pilar Gonzalo, María Jesús Andrés-Manzano, Beatriz Dorado, Ignacio Benedicto, Vicente Andrés

**Affiliations:** 1 Laboratory of Molecular and Genetic Cardiovascular Pathophysiology, Centro Nacional de Investigaciones Cardiovasculares (CNIC), Melchor Fernández Almagro 3, 28029 Madrid, Spain; 2 CIBER en Enfermedades Cardiovasculares (CIBER-CV), Instituto de Salud Carlos III, Madrid, Spain; 3 Centro de Investigaciones Biológicas Margarita Salas (CIB-CSIC), 28040 Madrid, Spain

**Keywords:** Hutchinson-Gilford progeria syndrome, Ion channels, Hypoxia, Vascular smooth muscle cells, Carotid artery, Coronary artery

## Abstract

Hutchinson-Gilford progeria syndrome (HGPS) is an extremely rare genetic disease caused by expression of progerin, a lamin A variant that is also expressed at low levels in non-HGPS individuals. Although HGPS patients die predominantly from myocardial infarction and stroke, the mechanisms that provoke pathological alterations in the coronary and cerebral arteries in HGPS remain ill defined. Here, we assessed vascular function in the coronary arteries (CorAs) and carotid arteries (CarAs) of progerin-expressing *Lmna*^*G609G/G609G*^ mice (G609G), both in resting conditions and after hypoxic stimulus. Wire myography, pharmacological screening, and gene expression studies demonstrated vascular atony and stenosis, as well as other functional alterations in progeroid CorAs and CarAs and aorta. These defects were associated with loss of vascular smooth muscle cells and overexpression of the K_V_7 family of voltage-dependent potassium channels. Compared with wild-type controls, G609G mice showed reduced median survival upon chronic isoproterenol exposure, a baseline state of chronic cardiac hypoxia characterized by overexpression of hypoxia-inducible factor 1α and 3α genes, and increased cardiac vascularization. Our results shed light on the mechanisms underlying progerin-induced coronary and carotid artery disease and identify K_V_7 channels as a candidate target for the treatment of HGPS.

**Supplementary Information:**

The online version contains supplementary material available at 10.1007/s11357-023-00808-3.

## Introduction

Hutchinson-Gilford progeria syndrome (HGPS) is an extremely rare disease (estimated prevalence, 1 in 18–20 million people; https://www.progeriaresearch.org/) characterized by premature aging, exaggerated cardiovascular disease (CVD), and death (average lifespan 14.6 years) [1-6]. Most HGPS patients carry a de novo heterozygous c.1824C > T (pG608G) point mutation in the *LMNA* gene [7, 8]. This mutation triggers use of an unusual 5´ splice donor site in exon 11 that removes 150 nucleotides from the *LMNA* mRNA, resulting in the synthesis of a mutant form of lamin A called progerin. Unlike mature lamin A, progerin remains permanently farnesylated and methylated at its carboxy terminus and accumulates in the nuclear envelope, causing severe nuclear structural and functional alterations [1, 4]. Remarkably, several studies have demonstrated low-level progerin expression during normal aging (reviewed in [4, 9]). Moreover, progeria features most of the hallmarks of normal aging, suggesting that progerin may contribute to some of the pathophysiological features commonly observed in the elderly population (e.g., cardiovascular alterations) [1, 4, 10]. Although the US Food and Drug Administration and the European Medicines Agency recently approved the use of lonafarnib (marketed as Zokinvy™) for the treatment of HGPS patients [11-13], there is still an urgent need for better targets and therapies to improve and eventually cure HGPS.

The main medical problem in HGPS is severe CVD, including accelerated atherosclerosis and vascular calcification and stiffness, which ultimately provoke myocardial infarction, stroke, or heart failure, the main causes of death in HGPS [4, 5]. Although HGPS patients show well-described alterations in the coronary arteries (CorAs) and carotid arteries (CarAs) [6, 10, 14-17], very little is known about the mechanisms that cause alterations in these vessels. For example, despite the well-known role of ion channels in the regulation of vascular smooth muscle cell (VSMC) plasma membrane depolarization and vessel contraction, their possible involvement in the vascular pathology of HGPS has not been explored in depth. In addition, there is some evidence suggesting cardiac hypoperfusion in HGPS animal models [18, 19]. In this study, we used homozygous *Lmna*^*G609G/G609G*^ (G609G) mice, a model characterized by the development of most progeroid features, including failure to thrive, lipodystrophy, bone abnormalities, and cardiovascular alterations [19-24] to characterize the CorAs, CarAs, and aorta and to explore the hypothesis that vascular dysfunction contributes to chronic cardiac hypoxia and consequently heart failure, myocardial infarction, and stroke in HGPS. Our results identify vascular atony, elevated markers of chronic hypoxia, marked VSMC loss, and overexpression of K_V_7 voltage-gated potassium channels in aorta, CorAs, and CarAs in G609G mice, suggesting vascular K_V_7 channels as a new therapeutic target in HGPS.

## Methods

### Data availability

The data from this study are available in the main article or in the online supplementary material. Data, analytical methods, and study materials will be made available to other researchers upon reasonable request for the purposes of reproducing the results or replicating the procedures (available from the authors’ laboratories).

### Study approval

All animal studies have been performed in accordance with the ethical standards laid down in the 1964 Declaration of Helsinki and its later amendments, and conformed to EU Directive 2010/63EU and Recommendation 2007/526/EC, enacted in Spanish law under *Real Decreto 53/2013*. All animal protocols were approved by the local ethics committees and the Animal Protection Area of the Comunidad Autónoma de Madrid (PROEX 050/18).

### Mice

Animal experiments were carried out on 17–19-week-old male and female G609G mice; at this age, the disease is advanced but has not reached a severity comparable to human endpoint criteria [19, 20]. Controls were age-matched wild-type littermates (WT). All mice were on the C57BL/6J genetic background and were reared and housed in accordance with institutional guidelines and regulations, under specific pathogen-free conditions at a constant temperature of 23 ± 1 °C, relative humidity 58%, and a 12-h dark/light cycle. Mouse health was monitored in a blinded manner at regular intervals throughout the study. G609G mice were housed with WT littermates to maintain the best housing conditions. For in vivo experiments, animals were anesthetized with 0.5–2% isoflurane in an inhalation chamber. Animals were euthanized in a CO_2_ chamber. The time of CO_2_ asphyxiation was determined as the time elapsed from the initiation of CO_2_ exposure until no breathing movement was detected by the observer.

### Wire myography analysis of vascular function

Aortic, carotid, and coronary artery function was assessed by wire myography [25-27]. Briefly, animals were sacrificed and the aorta, CarAs, and hearts were excised, washed, and preserved in Krebs–Henseleit solution (KHS: 115 mM NaCl, 2.5 mM CaCl_2_, 4.6 mM KCl, 1.2 mM KH_2_PO_4_, 1.2 mM MgSO_4_, 25 mM NaHCO_3_, 11.1 mM glucose, and 0.01 mM EDTA). The arteries were gently cleaned of surrounding fat (including the vagal nerve in the case of CarAs) and connective tissue, and were cut into ~ 2-mm-long segments. The left and right CorAs (LCorA and RCorA) were carefully dissected, cleaned of cardiac tissue, and cut into ∼2-mm-long segments. Arterial rings were mounted on two tungsten wires (40-µm diameter for aortas; 25-µm diameter for CarAs and CorAs) in a wire myograph system (620M, Danish Myo Technology A/S, Hinnerup, Denmark) and immersed in 37 °C KHS with constant gassing (95% O_2_ and 5% CO_2_). Optimal vessel distension was determined by normalization using the Laplace equation [tension = (pressure × radius)/thickness] to calculate the position at which the tension was equivalent to an intraluminal pressure of 100 mmHg (L100) [25-27]. Vessels were then set up to the optimal tension (physiological distension, 0.9 × L100).

After equilibration for 30 min, vasoconstriction was studied by exposing the rings either to 80 mM KCl followed by 1 µM phenylephrine or to increasing concentrations of the K_V_7 channel blocker XE-991 (from 10 nM to 10 µM; Cat. No. 2000, Tocris). Consecutive treatments were separated by extensive washes and a stabilization period of at least 30 min.

### Response to hypoxia

The effects of acute hypoxia on CarAs and CorAs were studied under resting conditions. To induce hypoxia, the organ chambers were wrapped in cling film and the gas mixture aerating the organ bath was switched from 95% O_2_, 5% CO_2_ to 95% N_2_, 5% CO_2_. This procedure routinely achieved an oxygen saturation of 2%-3%, measured with an HI2040-02 Multiparametric EDGE dissolved-oxygen meter (Hanna Instruments). Responses to reperfusion were assessed after restoring the initial conditions of 95% O_2_, 5% CO_2_.

### Echocardiography

Transthoracic echocardiography was performed by a blinded expert operator from the CNIC Advanced Imaging Unit using a high-frequency ultrasound system (Vevo 2100, Visualsonics Inc., Canada) equipped with a 40-MHz linear probe. Two-dimensional echography was performed at a frame rate above 230 frames/s. Chest hair was removed with hypoallergenic depilatory cream, and mice were lightly anesthetized with 0.5–1.5% isoflurane in oxygen at a flow rate of 1.5 L/min. Mice were placed in a supine position on a heated platform, and warmed ultrasound gel was used to maintain normothermia. Mice were continuously monitored by base apex electrocardiography (ECG). Images were transferred to a computer and analyzed offline with the Vevo 2100 Workstation software. Blood flow in the CarAs and CorAs was measured in pulsed wave Doppler mode. Coronary flow reserve (CFR) was assessed after i.p. administration of a single 2 mg/kg dose of isoproterenol.

### Electrocardiography

Mice were anesthetized with 0.5–1.5% isoflurane in oxygen, inhaled through a face mask. To avoid interference from circadian variations, all ECG traces were recorded in the morning. ECG electrodes were inserted subcutaneously in all four limbs. ECG recordings were acquired at 2 kHz using a MP36R data acquisition workstation (Biopac Systems) and exported with AcqKnowledge software (Biopac Systems) for manual analysis. Lead II was selected for the study, since this signal was more stable in most experiments, thus allowing more robust wave identification. Ischemia and heart failure ECG morphologies were assessed by observers blinded-to-genotype according to previous studies [28].

### Survival of isolated cardiomyocytes in the hypoxia chamber

The procedure was adapted from Macías et al. [19] and Garcia-Prieto et al. [29]. Briefly, after CO_2_ asphyxiation, mice were placed in the supine position, and the ventral thoracic region was wiped with 70% alcohol. The heart was quickly removed and incubated at room temperature (RT) in Ca^2+^-free perfusion buffer (PB: 113 mM NaCl; 4.7 mM KCl; 0.6 mM KH_2_PO_4_; 0.6 mM Na_2_HPO_4_; 1.2 mM MgSO_4_·7H_2_O; 12 mM NaHCO_3_; 10 mM KHCO_3_; 0.032 mM phenol red; 10 mM HEPES; 30 mM taurine; 5.5 mM glucose; 10 mM 2,3-butanedione-monoxime; pH 7.4). Fat was removed, and the heart was cannulated through the ascending aorta and mounted on a modified Langendorff-perfusion apparatus. The heart was then retrogradely perfused with PB (1 mL/min) for 5 min at 37 °C. To isolate cardiomyocytes, the heart was perfused with digestion-buffer (DB: PB supplemented with liberase™ (0.2 mg/mL; Cat. No. 5401127001, Sigma-Aldrich), 5.5 mM trypsin (2.5%; Cat. No. 15090046, Gibco), and 12.5 µM CaCl_2_) for 20 min at 37 °C. At the end of the enzymatic digestion, both ventricles were isolated and gently disaggregated in 3 mL DB. The resulting cell suspension was filtered through a 200-µm sterile mesh (SEFAR-Nitex) and transferred for enzymatic inactivation to a tube containing 10 mL stopping buffer 1 (SB-1: PB supplemented with 10% v/v fetal bovine serum (FBS) and 12.5 µM CaCl_2_). After gravity sedimentation for 20 min, the supernatant was removed, and cardiomyocytes were resuspended in stopping buffer 2 (SB-2: PB supplemented with 5% v/v FBS) for a further 20 min. Cardiomyocytes were reloaded with Ca^2+^ by sequential incubation in SB-2 containing 0.112 mM and then 1 mM CaCl_2_. At each step, cells were resuspended and allowed to settle for 15 min followed by removal of the supernatant, contributing to the purification of the cardiomyocyte suspension. Cardiomyocyte cultures were washed and stabilized for 30 min at 37 °C with KHS before the induction of hypoxia [29]. Hoechst 33342 (1 µg/mL; Cat. No. B2261, Sigma-Aldrich) and propidium iodide (1 µg/mL; Cat. No. 537059, Sigma-Aldrich) were added to identify cells and to assess cell viability, respectively. Hypoxia was induced by placing cells in a H35 Hypoxystation chamber (Don Whitley Scientific Limited, UK). The buffer used during hypoxia was pre-equilibrated to 1% O_2_ for 2 h before use. Fluorescence images were acquired with a Nikon Time-lapse confocal microscope at time 0 and after 1 h of hypoxia. A blinded observer analyzed an average of 300 rod-shaped cells/well from 5 independent experiments using ImageJ Fiji software. Cell death, indicated by internalization of red propidium iodide fluorescence, was expressed as a percentage of the total number of cardiomyocytes (blue fluorescence due to Hoechst 33342 staining).

### Mouse survival studies

Chow‐fed mice were weighed at the beginning of the experiment and inspected daily for health and survival before treatment. Mice received i.p. injections of isoproterenol starting at 16 weeks of age (2 mg/kg/day). Animals that met humane endpoint criteria were sacrificed, and the deaths were recorded for the survival curve analysis.

### In vivo vessel lumen assay

Vessel permeability was examined in anesthetized mice essentially as described [30]. Briefly, the mice were injected intravenously (retro-orbital administration) with 100 µl of Alexa488-labeled wheat germ agglutinin (WGA; 1 mg/ml; Cat. No. W11261, Thermo Scientific). After 30 min, mice were sacrificed, and hearts were extracted and processed for whole-mount staining.

### Histology and immunofluorescence studies

Mouse hearts were fixed in 4% formaldehyde in PBS, dehydrated in 70% ethanol, and embedded in paraffin. For the structural study, 5-µm heart sections were deparaffinized and rehydrated. For immunofluorescence, antigens were retrieved by incubation in 0.05% Tween-20 and 10 mmol/L sodium citrate buffer (pH 6). Samples were then blocked and permeabilized for 1 h at RT in PBS containing 0.3% Triton X-100, 5% BSA (Cat. No. A7906, Sigma-Aldrich), and 5% normal goat serum (Cat. No. 005–000-001, Jackson ImmunoResearch) and incubated overnight at 4 °C with Cy3-labeled anti-smooth muscle actin (SMA: Cat. No. C6198, Merck, 1/500). Samples were then incubated for 2 h with Alexa488-labeled WGA (1/100) and Hoechst 33342 (1/1000) and mounted in Fluoromount-G imaging medium. Images were acquired with a LSM 880 Carl Zeiss confocal microscope and analyzed by genotype-blinded observers using ImageJ Fiji software. The percentage of the CorA surface positive for SMA was quantified in 2–3 ventricular areas per heart. Vascular ramification (branches) and volume were quantified using an ImageJ Fiji macro customized by the CNIC Microscopy Unit and based on the skeletonization of hearts perfused with WGA in vivo.

### RNA isolation and mRNA expression analysis

Total heart RNA was extracted from powdered mouse tissue samples by resuspension in TriReagent Solution (Cat. No. AM9738, Thermo Fisher) and homgenization with stainless steel 5-mm beads (Cat. No. 69989, Qiagen) in a TissueLyser LT device (Qiagen), followed by ethanol precipitation according to the manufacturer’s instructions. RNA was extracted from CarAs using the miRNeasy Micro Kit (Cat. No. 217084, Qiagen), and samples were treated with DNase I (Cat. No. 79254, Qiagen). RNA concentration was quantified in a NanoDrop ND-1000 spectrophotometer (Wilmington), and 1–2 µg were transcribed to cDNA using the High-Capacity cDNA Reverse Transcription Kit (Cat. No. 4368814; Applied Biosystems, Foster City, CA USA). Quantitative real-time PCR (qPCR) was performed using the primers shown in Table 1 and the Power SYBR Green PCR Master Mix (Cat. No. 4368702, Applied Biosystems) in a C1000 Touch Thermal Cycler (Bio-Rad). Housekeeping genes for normalization were *Gapdh* and *Tbp*. For *Kcnq* studies in heart tissue, *Acta2* was included as a VSMC-specific gene. Fold changes were determined using the 2^-ΔΔCT^ method.

**Table 1:** Primer sequences

Gene	Forward (5′ → 3′)	Reverse (5′ → 3′)
*Gapdh*	GGTTGTCTCCTGCGACTTCA	TAGGGCCTCTCTTGCTCAGT
*Tbp*	CCCCTTGTACCCTTCACCAAT	GAAGCTGCGGTACAATTCCAG
*18S*	CGCAGAATTCCCACTCCCGACCC	CCCAAGATCCAACTACGAGC
*Acta2*	AAGAGGAAGACAGCACAGCC	AGCGTCAGGATCCCTCTCTT
*Kcnq1*	TTCGCCACATCAGCTATCAG	AATGTACAGGGTGGTGATCAG
*Kcnq2*	GTTCATCTACCACGCCTACG	CACCGAATACCACGATAGTCAC
*Kcnq3*	GTCAGCATTTACCTACCCATCC	GTCCAGTTTCTTCCCCATGTC
*Kcnq4*	GGAGGCAGTGGATGAAATCAG	AGTAGAAGCCCAGCAGCA
*Kcnq5*	TCATCCTTCCTTGTCTATCTTGTG	AGTCTTCCCAGCCACGTTA
*Hif1a*	GAATGCTCAGAGGAAGCGAAA	ACAGTCACCTGGTTGCTGCA
*Hif3a*	CAAGCGGTCCCCAAGACTAG	TCATGAGAATGCACGAGGGG

### Bulk RNAseq data analysis

We analyzed bulk RNAseq data corresponding to hearts from 16-week-old G609G mice and 12-, 52-, and 104-week-old wild-type mice that were derived from a previous report [31] and were deposited in the Gene Expression Omnibus database (GSE124005). Reads were pre-processed with cutadapt and fastqc, and aligned against the mouse transcriptome (version GRCm38.99) using RSEM. Mutidimensional scaling (MDS) plot was generated with the plotMDS function from EdgeR, using the biological coefficient of variation (BCV) method. Samples SRR8352396 (52-week-old WT mice) and SRR8352401 (104-week-old WT mice) behaved as outliers in the MDS plot and were discarded before further analysis. Differential gene expression between G609G and 12-week-old wild-type mice was analyzed with Limma using 13,291 genes with at least 1 copy per million (cpm) in three or more samples. We set the statistical significance threshold at Benjamini–Hochberg corrected *p* < 0.05. Gene ontology analysis was carried out with the DAVID software [32] using differentially expressed genes with fold change > 1.25 and <  −1.25. Predicted upstream transcriptional regulators were identified using the Ingenuity Pathway Analysis software (Qiagen).

### Statistical analysis

Statistical analyses were conducted using Prism-5 and Prism-7 software. Between-genotype comparisons were made by unpaired two-tail Student *t*-test. Unless otherwise stated, comparisons between more than two groups were by one- or two-way ANOVA with Tukey’s correction. Grubbs’ test was performed to exclude outliers from some data sets. Unless otherwise stated, N represents the number of mice, and *n* is the number of samples used in each experimental set. Kaplan–Meier survival curves were compared by the log-rank (Mantel-Cox) test. Data are expressed as mean ± SEM, and differences were considered significant at *p* < 0.05.

## Results

### Atony and stenosis in the carotid and coronary arteries of G609G mice

**Fig. 1:**
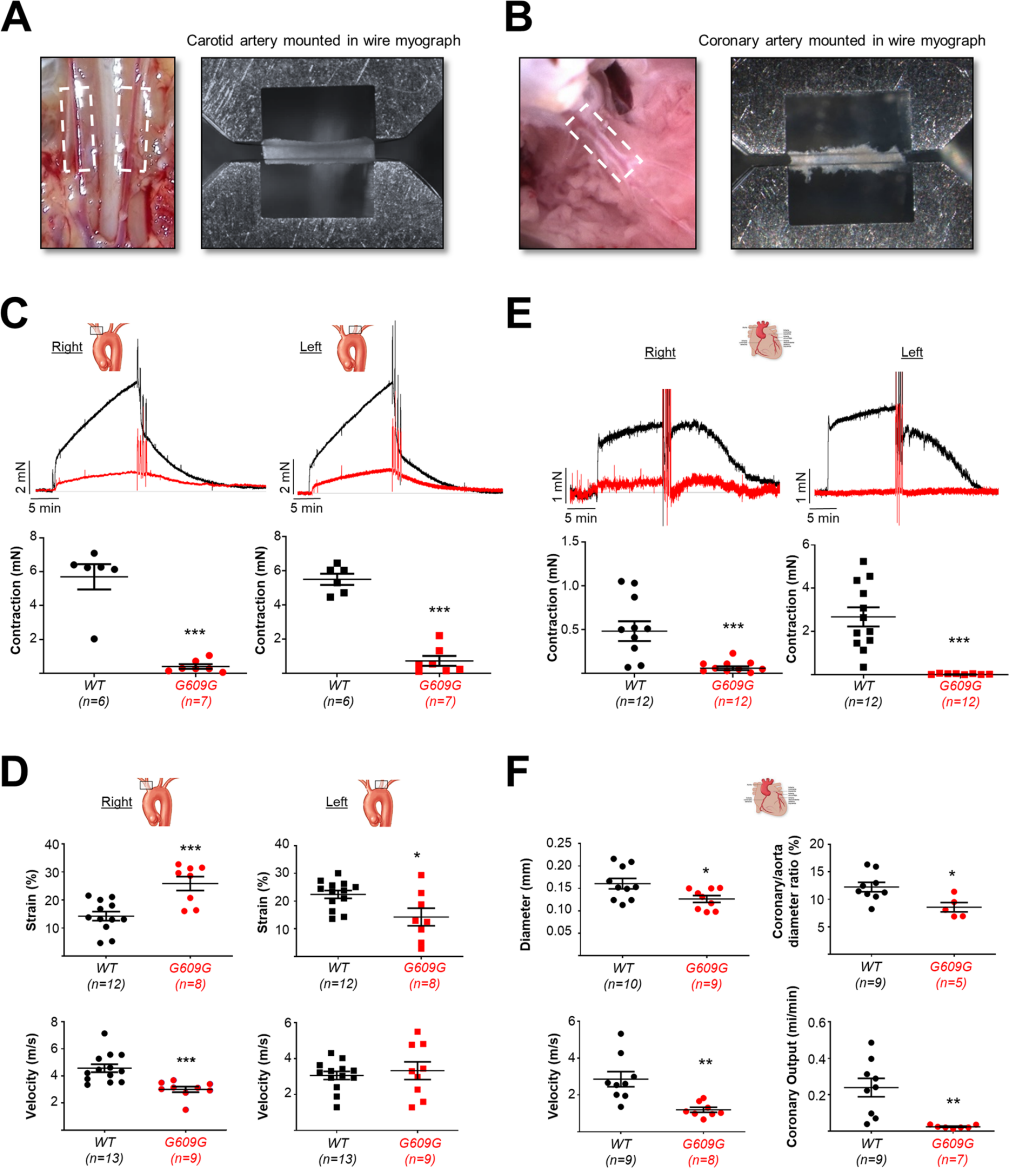
Progerin expression results in carotid and coronary atony and stenosis. The contractile properties of carotid (**A**,** C**, **D**) and coronary (**B**, **E**, **F**) arteries from wild-type (WT) and G609G mice were analyzed ex vivo by wire myography (**C** and **E**) and in vivo by echocardiography (**D** and **F**) (examples in Supplementary Videos S1 and S2). **A**, **B** Examples of a carotid artery (CarA, **A**) and a coronary artery (CorA, **B**) mounted in the wire myograph system. **C** Strength of contraction induced by 80 mM KCl in right and left CarAs, showing representative myograph recordings (top) and quantification (bottom). **D** Echocardiography analysis of innominate artery (right CarA) and left CarA to assess the percentage of strain (top) and blood velocity (bottom). **E** Strength of contraction induced by KCl 80 mM in coronary arteries (CorAs) showing representative wire myograph recordings (*top*) and quantification (bottom). **F** Echocardiography analysis of septal CorAs, assessing coronary diameter (top) and coronary blood velocity and output (bottom). Statistical differences were analyzed by two-tailed *t*-test. *p < 0.05; **p < 0.01; ***p < 0.001

We performed ex vivo wire myography to analyze the contractile function of the right and left carotid arteries (RCarAs and LCarAs, respectively) and coronary arteries (RCorAs and LCorAs) isolated from WT and G609G mice (Fig. 1A, B). RCarAs and LCarAs from G609G mice showed a significantly reduced capacity to contract in response to 80 mM KCl (Fig. 1C). We next performed in vivo vascular ultrasound to examine strain and blood flow velocity in CarAs. Progeroid CarAs showed opposite alterations in strain relative to WT controls, with strain elevated in the RCarA and diminished in the LCarA (Fig. 1D, upper graphs), whereas blood velocity was diminished in the RCarA but not significantly affected in the LCarA (Fig. 1D, bottom graphs). Similarly, progeroid CorAs mounted in the wire myograph exhibited almost no contractility when exposed to 80 mM KCl (Fig. 1E). In addition, echocardiography experiments revealed that G609G CorAs had smaller vessel diameter (absolute values and relative to aorta diameter) and lower blood velocity (Fig. 1F). In combination with the bradycardia exhibited by this model (see below and refs [19, 33]), the lower blood velocity in progeroid CorAs markedly decreased coronary output (Fig. 1F).

### G609G mice exhibit cardiac hypoperfusion under stress conditions

**Fig. 2:**
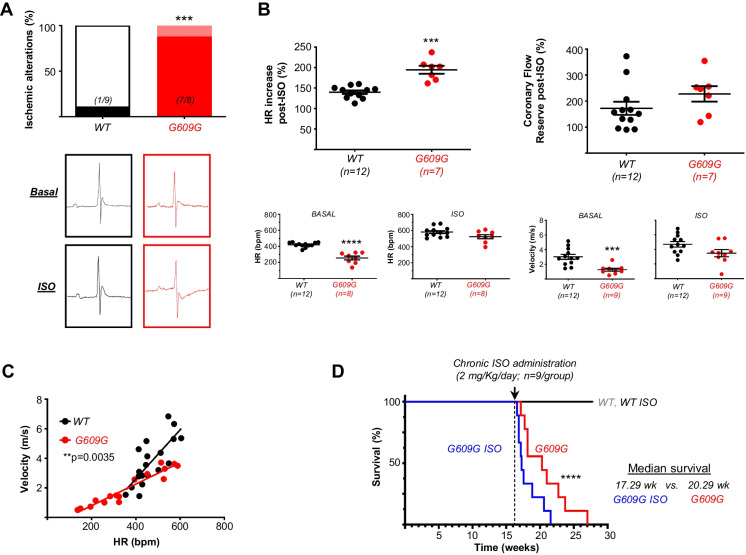
Coronary artery dysfunction in G609G mice. **A** Effect of acute isoproterenol administration (2 mg/kg, single dose) on the electrocardiographic (ECG) pattern. The graph shows the incidence of ECG alterations indicating cardiac hypoperfusion. **B** Effects of acute isoproterenol administration (2 mg/kg, single dose) on heart rhythm (HR; left) and coronary flow reserve (CFR; right) assessed by echocardiography. **C** Correlation between velocity and HR during the experiment shown in **B**. **D** Kaplan–Meier survival curve (n = 9/group) in WT and G609G mice chronically treated with vehicle or isoproterenol (2 mg/kg/day). Differences were analyzed by the Fisher exact test (**A**), two-tailed Student *t*-test (**B**), Pearson r test (**C**), and Mantel-Cox test (**D**). **p < 0.01; ***p < 0.001; ****p < 0.0001

To assess the ability of the progeroid heart to adapt to stress, we analyzed cardiac function in mice challenged with isoproterenol (Fig. 2, Supplemental Videos S1 and S2). After isoproterenol challenge, ECG recordings showed alterations compatible with cardiac hypoperfusion (ischemia or infarction) [28], with 88% of G609G mice showing a prolonged QRS interval and a JT segment with a positive, large T wave and entirely negative and inseparable J and T waves, whereas this pattern was seen in only 11% of WT mice (Fig. 2A). Echocardiography analysis showed isoproterenol-induced increases in heart rhythm (HR) and CFR in both WT and G609G mice, but these changes were more pronounced in progeroid hearts (Fig. 2B, upper graphs) despite their lower baseline values (Fig. 2B, lower insets). Consequently, the correlation between HR and CFR had a significantly shallower slope in G609G mice (Fig. 2C). To investigate the response to chronic stress, we treated mice with a daily dose of isoproterenol (2 mg/kg/day) starting at 16 weeks of age to provoke cardiac hypertrophy [34]. Consistent with the observed coronary atony and cardiac hypoperfusion, chronic isoproterenol exposure decreased the survival of G609G mice but did not affect the survival of WT mice (Fig. 2D).

### The cardiac phenotype of G609G mice is consistent with chronic hypoxia

**Fig. 3:**
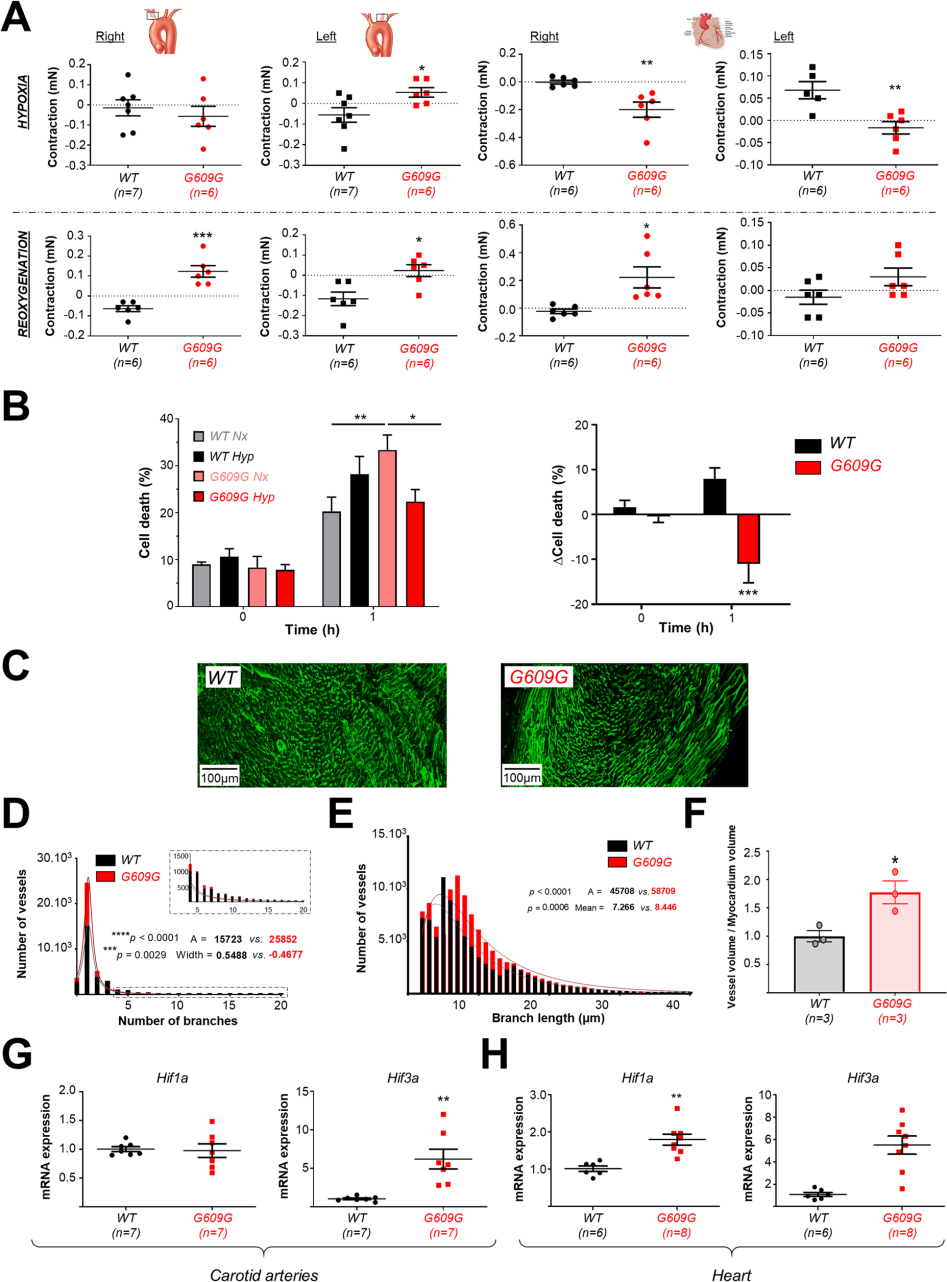
The cardiac phenotype of G609G is consistent with chronic hypoxia. **A** Contraction induced by hypoxia (top) and reoxygenation (bottom) in CarAs (left) and CorAs (right).** B** Cell death in isolated cardiomyocytes assessed as the propidium iodide:Hoechst 33342 ratio (left) and the variations in cell death after hypoxic stimulus (1% O_2_) (right). **C**–**F** Coronary irrigation after in vivo perfusion with wheat germ agglutinin (WGA). The graphs show the frequencies (number of vessels) of branch number (**D**) and branch length (**E**) and the normalized vessel:myocardium volume ratio (**F**). Distributions are shown in histograms with bins size of 1 branch (**D**) and 1 µm for length (**E**). **G**, **H** qPCR analysis of *Hif1a* and *Hif3a* mRNA in CarAs (**G**) and heart (**H**) in WT and G609G mice. *Gapdh* and *Tbp* mRNA levels were used as housekeeping genes for normalization, and results are presented relative to WT expression. Differences were analyzed by two-tailed Student *t*-test (**A**, **G**, **H**) and One-way ANOVA test (**B**, **D**, **E**). *p < 0.05; **p < 0.01; ***p < 0.001

The alterations described above are likely to reduce tissue irrigation, thereby lowering the oxygen supply and producing chronic hypoxia [35, 36]. To confirm this, we first performed ex vivo wire myography experiments to evaluate the response of vessels to changes in oxygen availability. Relative to WT vessels, progeroid CarAs and CorAs showed altered responses to both hypoxia and reoxygenation, indicating relative vascular dysfunction (Fig. 3A). We also analyzed cell death in primary cardiomyocyte cultures submitted to 1 h of hypoxia in vitro. Surprisingly, while WT cardiomyocytes exhibited increased cell death in hypoxia compared with normoxia, hypoxia was associated with less cell death in G609G cardiomyocytes (Fig. 3B).

**Fig. 4:**
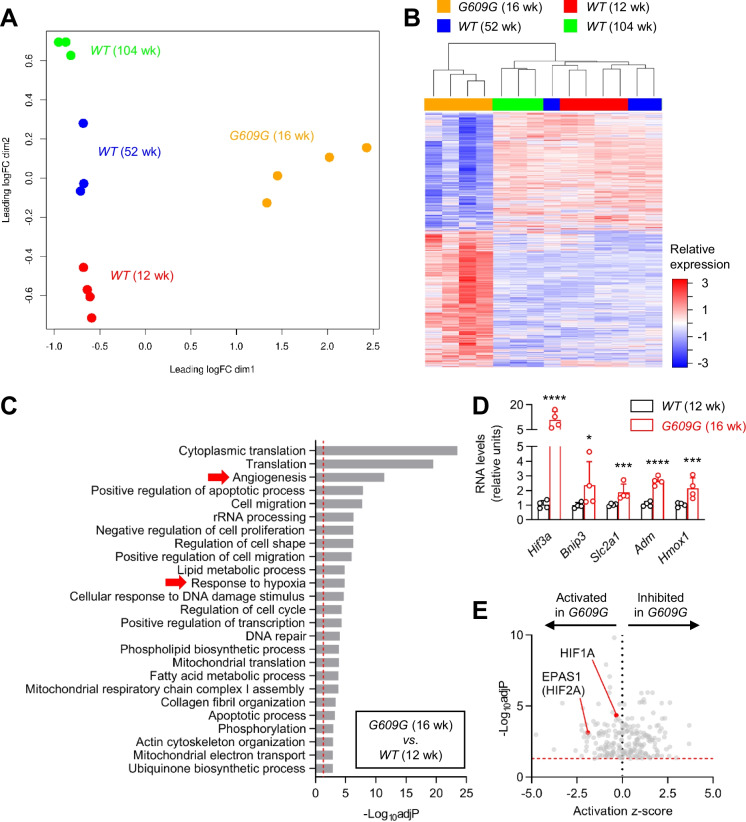
Bulk RNAseq data analysis suggests increased hypoxia and angiogenesis in the heart of G609G mice. We analyzed bulk RNAseq data in hearts from 12-, 52-, and 104-week-old WT and 16-week-old G609G mice (2 males and 2 females) obtained from the Gene Expression Omnibus database (GSE124005) [31]. Multidimensional plot (**A**) and heatmap (**B**) show a clear separation between WT and G609G. **C** Gene ontology analysis showing the 25 most significant categories altered in 16-week-old G609G vs. 12-week-old WT mice, including genes related to hypoxia and angiogenesis. **D** Hypoxia-induced genes are expressed at higher level in hearts from 16-week-old G609G mice vs. 12-week-old WT mice. Benjamini-Hochberg adjusted *p* < 0.0001 (****), *p* < 0.001 (***), and *p* < 0.05 (*).** E** Ingenuity Pathway Analysis software predicts the activation of the hypoxia-induced factors HIF1A and EPAS1 (HIF2A) in hearts from 16-week-old G609G vs. 12-week-old WT mice. The red dotted line in **C** and **E** indicates Benjamini-Hochberg adjusted *p* = 0.05

These results suggest a state of chronic hypoxia in the progeroid heart, which would likely trigger angiogenesis and increase cardiac vascularization [37, 38]. To investigate this hypothesis, we examined the length and structure of the coronary arteries and heart vessels by perfusing with WGA in vivo (Fig. 3C and Supplementary Video S3). G609G hearts had a higher number of branched vessels (Fig. 3D), longer vessels (with both unimodal and right-skewed patterns of distribution) (Fig. 3E), and a greater vessel volume relative to the myocardium (Fig. 3F). In addition, qPCR analysis of canonical hypoxia-inducible factors (HIFs) revealed a significantly higher level of both *Hif1a* and *Hif3a* expression in G609G hearts and of *Hif3a* in G609G CarAs (Fig. 3G, H). G609G mice also took longer to die at euthanasia by CO_2_ asphyxiation (Supplemental Fig. S1). Moreover, bioinformatics analysis of previously published bulk RNAseq data [31] showed gene expression alterations consistent with increased hypoxia and angiogenesis in G609G hearts compared to WT controls (Fig. 4). Collectively, these observations suggest a state of adaptation to chronic hypoxia in G609G mice.

### Dysregulation of Kv7 ion channels in progeroid arteries

**Fig. 5:**
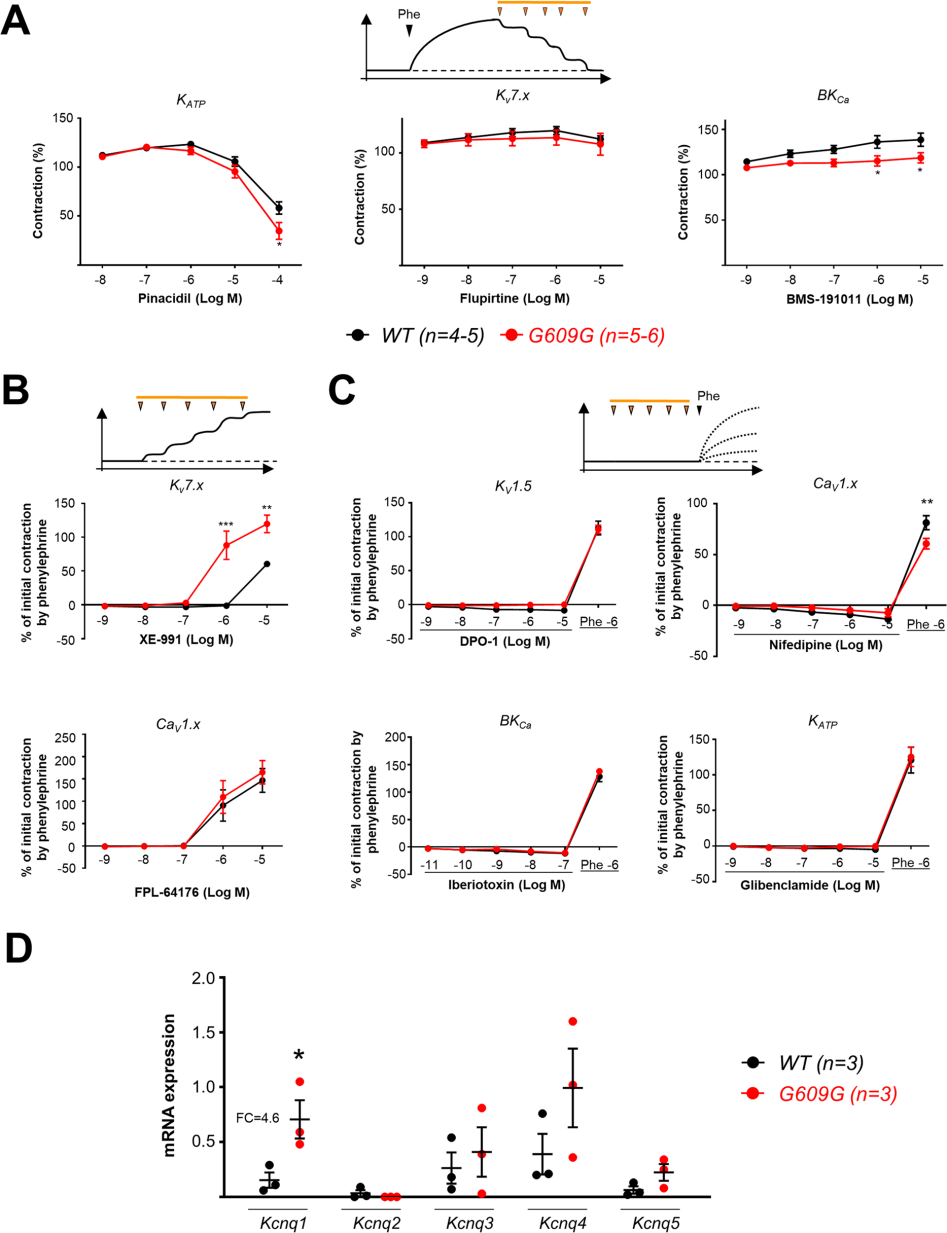
Kv7 is the main ion channel altered in the G609G aorta. **A**–**C** Pharmacological screening of ion channel modulators potentially involved in the mechanism of arterial dysfunction in G609G mice. Panels show concentration–response curves evaluating** A** vasodilation (pinacidil for K_ATP_, flupirtine for the K_V_7.x family, and BMS-191011 for BK_Ca_), **B** vasoconstriction (XE-991 for K_V_7.x family and FPL-64176 for the Ca_V_1.x family), and **C** modulators of vasoconstriction mediated by phenylephrine (DPO-1 for K_V_1.5, nifedipine for the Ca_V_1.x family, iberiotoxin for BK_Ca_, and glibenclamide for K_ATP_). Experimental procedures are schematized above the graphs. **D** Analysis of *Kcnq1-5* mRNA expression in WT and G609G aorta. Expression was normalized to 18S mRNA levels. Differences were analyzed by two-way ANOVA (**A**–**C**) and two-tailed Student *t*-test (**D**). *p < 0.05; **p < 0.01; ***p < 0.001

Vascular contraction begins with the depolarization of VSMCs, a process mediated directly by ion channels present in the VSMC plasma membrane. We hypothesized that alterations in ion channels would contribute to progerin-mediated VSMC dysfunction, in line with previous observations in HGPS cardiomyocytes [19]. To distinguish between the activities of different ion-channel families, we performed ex vivo wire myography studies to examine the reactivity of aortic rings (because of its larger size compared with CarAs and CorAs, working with aorta allowed us maximizing the number of drugs tested, thus minimizing the number of mice). Rings were treated with a battery of compounds classified as follows: potassium channel activators, as potential vasodilators (Fig. 5A); a potassium channel blocker and a calcium channel activator, as potential vasoconstrictors (Fig. 5B); and potassium channel activators and a calcium channel inhibitor, as potential contraction modulators (Fig. 5C). We found no significant between-genotype differences in aortic vasodilation in response to potassium channel activators (Fig. 5A). Among all the potassium channel blockers tested that induce vasoconstriction (Fig. 5B and C), the only one to show a between-genotype effect on aortic rings was the K_V_7 channel blocker XE-991, which induced stronger contraction at much lower concentrations in progeroid aorta than in WT aorta. qPCR analysis revealed significantly higher expression of *Kcnq1* in G609G aortas (fold difference = 4.6) but no between-genotype differences in other Kv7 family members (Fig. 5D). These findings suggest that progerin-dependent *Kcnq* overexpression contributes to the functional differences between WT and progeroid aortas observed in wire myography experiments.

**Fig. 6:**
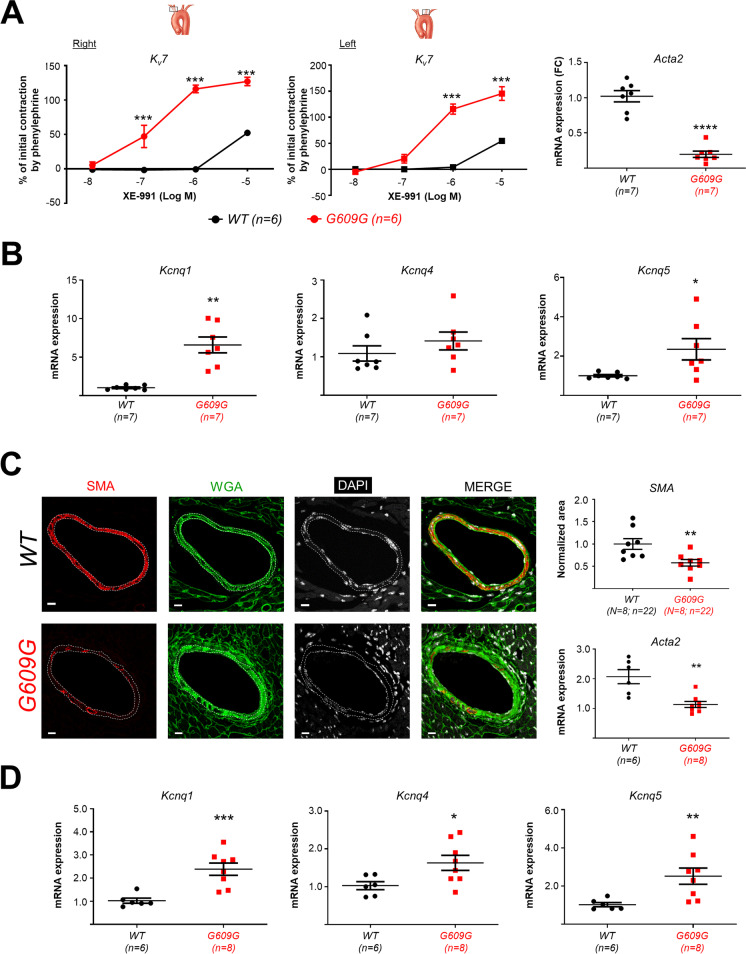
*Kcnq* overexpression in carotid and coronary arteries of G609G mice. **A** Left panel pair, XE-991-induced contraction in CarAs relative to initial contraction induced by 1 µM phenylephrine. Right, *Acta2* mRNA expression. *Gapdh* and *Tbp* mRNA levels were used as housekeeping genes for normalization, and results were represented relative to WT expression levels (**B**) *Kcnq1, Kcnq4,* and *Kcnq5* mRNA expression in CarAs. *Acta2* mRNA levels were used as housekeeping genes for normalization. Results are expressed relative to WT. **C** Transverse sections of WT and G609G coronary arteries showing immunofluorescent staining for smooth muscle actin (SMA) together with wheat germ agglutinin (WGA) and DAPI fluorescent signals (scale bar, 10 μm). The graphs show quantification of SMA area and *Acta2* mRNA expression. *Gapdh* and *Tbp* mRNA levels were used as housekeeping genes for normalization. **D** mRNA expression of *Kcnq1, Kcnq4, and Kcnq5* in CorAs*. Acta2* was mRNA levels were used as housekeeping genes for normalization. Differences were analyzed by two-tailed Student *t*-test with the exception of the wire myography studies shown in **A**, which were analyzed by two-way ANOVA. *p < 0.05; **p < 0.01; ***p < 0.001; ****p < 0.0001

Studies in CarAs revealed similarly stronger contraction in right and left progeroid CarAs relative to WT arteries at lower XE-991 doses (Fig. 6A). Moreover, G609G CarAs showed significantly higher mRNA expression of *Kcnq1* and *Kcnq5* (Fig. 6B). In marked contrast, G609G CorAs were unable to contract in response to several drugs, including serotonin (5-hydroxytryptamine), U-46619, phenylephrine, and endothelin-1 (data not shown). These alterations are consistent with a pronounced loss of VSMCs, as previously described in other vascular beds and progeria models [18, 33], which could in part explain the vascular atony observed in G609G CorAs. VSMC loss was confirmed by immunofluorescence, which revealed a ≈ 50% reduction in the expression of the VSMC-specific marker SMA in the media of G609G CorAs, accompanied by elevated extracellular matrix deposition (indexed by intracellular WGA staining) (Fig. 6C). Accordingly, *Acta2* mRNA expression was significantly lower in progeroid CorAs (Fig. 6C, lower graph). We also found higher mRNA expression of *Kcnq1*, *Kcnq4*, and *Kcnq5* in G609G CorAs (Fig. 6D). Moreover, the *Kcnq1* gene was significantly overexpressed in the previously published heart bulk RNAseq data (fold change = 1.62; *p* = 0.00049) [31]. Altogether, these data identify *Kcnq1* as a potential target to improve vascular function in progeria.

## Discussion

HGPS is an extremely rare genetic disease characterized by premature aging and death during adolescence, predominantly from myocardial infarction, stroke, or heart failure [1, 2, 4, 5, 12]. *LMNA* mutations are associated with changes in nuclear envelope structure and function that cause cytoskeletal defects in cell structure, gene expression, and protein trafficking and function [19, 39-43]; however, the mechanisms underlying progerin-induced alterations in CarAs and CorAs remain largely unknown. The results presented here show that progerin-expressing G609G mice exhibit severe dysfunction in both these vascular territories, including atony and stenosis and abnormal responses to hypoxia/reoxygenation. These changes are associated with chronic hypoxia indexed by increased expression of *Hif1a* and *Hif3a* genes, pronounced VSMC loss, and overexpression of K_V_7 voltage-gated potassium channels. Further studies are warranted to establish mechanistic links between progerin-related pathomechanisms and the observed alterations in progeroid arteries. These studies will be challenging due to the many cellular processes altered by progerin expression, including higher-order chromatin organization, DNA replication, DNA damage repair, gene transcription, and signal transduction [1, 4, 9], as well as the physiological relevance of Kv7 channels in the nervous system [44, 45].

Progerin expression significantly diminished ex vivo contractility in CarAs and CorAs, caused structural alterations, and reduced blood-flow velocity through these vessels. These alterations in vascular function are consistent with myocardial infarction, stroke, or heart failure, the main causes of death in HGPS patients [4, 5]. Interestingly, progerin expression increases with age in a subset of CorAs in non-HGPS individuals [6], suggesting that this aberrant lamin A variant promotes common cardiovascular alterations in both premature and physiological aging.

Progerin-induced vascular dysfunction can translate into a state of hypoperfusion-mediated chronic hypoxia in the progeroid heart. In the present study, we detected increased expression of members of the HIF pathway, mainly *Hif3a*, which undergoes rapid upregulation in the hypoxic heart [46] and has been linked to human end-stage heart failure [47]. Chronic activation of the HIF pathway appears to be maladaptive, contributing to cardiac degeneration and progression to heart failure [48], an observation consistent with the ventricular wall thinning we detected in G609G mice (Fig. 3D and Supplemental Video S3) and the interventricular septum reported previously in this mouse model [19]. These findings might help to explain, at least in part, the characteristic cardiac metabolic, structural, and electrophysiological alterations described in G609G mice [19, 49]. Moreover, the higher vascularization in G609G hearts might be a result of increased angiogenesis, a known consequence of chronic hypoxia [37, 38] that could be triggered as a compensatory mechanism of bradycardia- and vascular dysfunction-mediated hypoperfusion. Chronic hypoxia is also consistent with hypoxia-induced cardioprotection, which could explain the superior survival of G609G cardiomyocytes under hypoxia in vitro, as well as the higher resistance of G609G mice to CO_2_ asphyxiation. All these findings are concordant with the fetal gene program proposed to enact compensatory protective mechanisms to preserve cardiac contractile function during hypoxia [50].

Despite the physiological response to minimize the consequences of progerin-induced hypoxia, the vessels of G609G mice are severely dysfunctional, resulting in hypoperfusion. Resting cardiac hypoperfusion is a feature of our HGPS minipig model [18] and is also compatible with the cardiac electrical alterations and repolarization abnormalities described in HGPS patients and animal models [18, 19, 33, 49]. In the present study, we detected electrocardiographic alterations and increased cardiac hypoperfusion in G609G mice challenged with acute isoproterenol stress. Moreover, chronic treatment with a dose of isoproterenol that was non-lethal in WT mice reduced the lifespan of G609G mice. This chronic isoproterenol treatment increases cardiac oxygen demand and induces cardiac hypertrophy [34], which should stimulate increased coronary blood flow to satisfy this demand. However, the response in G609G CorAs was blunted, with only a mild increase in blood flow velocity. This may result from the observed VSMC loss, extracellular matrix deposition, and increased expression of K_V_7 channels, which play a key role in the control of CorA reactivity and responses to hypoxia [51-53]. Based on these results, we propose that hyperpolarization mediated by K_V_7 channel overexpression in progeroid vessels would result in the inactivation or dysfunction of voltage-gated calcium channels, leading to autophagy and apoptosis [54] and therefore loss of contractility. This vascular dysfunction would result in hypoperfusion and chronic hypoxia. These alterations, when combined with the bradycardia and the elevated number of circulating platelets in G609G mice (not shown) and HGPS patients [2, 22, 49], would create a ‘perfect storm’ for triggering myocardial infarction, stroke, and heart failure, the main causes of death in HGPS patients.

Progerin has a broad pattern of expression and has deleterious effects in many organs; however, we have demonstrated that restricting its expression to VSMCs and cardiomyocytes is sufficient to provoke CVD and reduce lifespan [22] and that suppressing its expression only in these cell types is sufficient to prevent CVD and normalize lifespan in progeroid mice [55]. The findings reported here reinforce the major role of vascular disease in HGPS and identify K_V_7 channels as a new candidate target for the treatment of this dramatic disease. Further studies are warranted to develop VSMC-specific tools in order to avoid undesired neurological disorders derived from targeting K_V_7 channels in the nervous system with available pharmacological approaches, which would result in spontaneous neuronal action potential firing [56-58].

### Supplementary Information

Below is the link to the electronic supplementary material.

Supplementary file 1 (MP4 3093 KB)

Supplementary file 2 (MP4 3354 KB)

Supplementary file 3 (MP4 11134 KB)

Supplementary file 4 (PDF 186 KB)
