# Supplementary material for: Coronary and carotid artery dysfunction and KV7 overexpression in a mouse model of Hutchinson-Gilford progeria syndrome
Source: GeroScience. 2023 May 26;46(1):867–84. doi: 10.1007/s11357-023-00808-3 (PMC10828489; doi:10.1007/s11357-023-00808-3)
Supplement: Supplementary file 4 — (PDF 186 KB) [file 11357_2023_808_MOESM4_ESM.pdf]

SUPPLEMENTARY MATERIAL

Coronary and carotid artery dysfunction and Kv7 overexpression in a mouse model of Hutchinson-Gilford progeria syndrome

Álvaro Macías[1], Rosa M. Nevado[1,2], Cristina González-Gómez[1,2], Pilar Gonzalo[1,2], María Jesús Andrés-Manzano[1,2], Beatriz Dorado[1,2], Ignacio Benedicto[1,3], Vicente Andrés[1,2,*]

[1] Centro Nacional de Investigaciones Cardiovasculares (CNIC), 28029 Madrid, Spain.
[2] CIBER en Enfermedades Cardiovasculares (CIBER-CV), Instituto de Salud Carlos III, Madrid, Spain.
[3] Centro de Investigaciones Biológicas Margarita Salas (CIB-CSIC), 28040 Madrid, Spain.

*** Corresponding author:**

Vicente Andrés, PhD
Molecular and Genetic Cardiovascular Pathophysiology
Centro Nacional de Investigaciones Cardiovasculares (CNIC)
Melchor Fernández Almagro 3, 28029 Madrid, Spain
Email: vandres@cnic.es
Telephone: +34-91 453 12 00 (Ext. 1502)
FAX: +34-91 453 12 65

Short title: Vascular dysfunction in progeria

Key words: Hutchinson-Gilford progeria syndrome; carotid artery; coronary artery; potassium channels; hypoxia

Author ORCIDs:

- *Álvaro Macías:* 0000-0002-9952-6947
- *Rosa M. Nevado*: 0000-0003-2341-3652
- *Pilar Gonzalo:* 0000-0001-8811-8369
- *Beatriz Dorado:* 0000-0002-1958-4558
- *Ignacio Benedicto:* 0000-0001-8081-1847
- *Vicente Andrés:* 0000-0002-0125-7209

SUPPLEMENTARY FIGURES AND VIDEOS

Supplementary Video S1. Echocardiography analysis of coronary artery dimensions

Supplementary Video S2. Echocardiography analysis of coronary flow

Supplementary Video S3. Cardiac vasculature in WT and G609G mice

Supplementary Figure S1. G609G mice are more resistant to euthanasia by CO_2 inhalation.

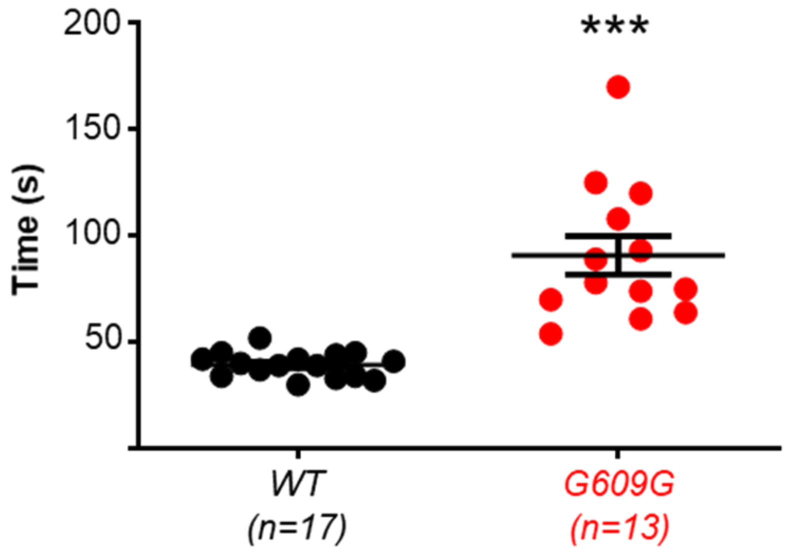

Supplementary Figure S1. G609G mice are more resistant to euthanasia by CO_2 inhalation. Time (in seconds) from the beginning of the CO_2 cycle to the death of the animal, defined as the absence of breathing movements. Differences were analyzed by two-tailed Student t-test. ***, $p<0.001$.
